# Spyke Viewer: a flexible and extensible platform for electrophysiological data analysis

**DOI:** 10.3389/fninf.2013.00026

**Published:** 2013-11-11

**Authors:** Robert Pröpper, Klaus Obermayer

**Affiliations:** ^1^Department of Software Engineering and Theoretical Computer Science, Neural Information Processing Group, TU BerlinBerlin, Germany; ^2^Bernstein Center for Computational NeuroscienceBerlin, Germany

**Keywords:** electrophysiology, python, data analysis, visualization, spike sorting, software, data sharing

## Abstract

Spyke Viewer is an open source application designed to help researchers analyze data from electrophysiological recordings or neural simulations. It provides a graphical data browser and supports finding and selecting relevant subsets of the data. Users can interact with the selected data using an integrated Python console or plugins. Spyke Viewer includes plugins for several common visualizations and allows users to easily extend the program by writing their own plugins. New plugins are automatically integrated with the graphical interface. Additional plugins can be downloaded and shared on a dedicated website.

## Introduction

The amount of data produced by electrophysiological experiments and neural simulations continues to grow. Accordingly, the complexity of analysis algorithms and infrastructure is increasing. Collaborations between research groups with different expertise are becoming the norm and the same data set is often analyzed by many scientists using diverse and dynamically evolving analysis methods.

This situation presents various challenges. Data sharing is difficult because electrophysiology data is very heterogeneous and no generally accepted standard formats exist yet. Over the years, recording equipment vendors and simulation software authors have defined specific data formats and many research groups have created their own proprietary formats. Analysis code is often written for a particular dataset and then discarded or rewritten for other data. As a consequence, algorithms developed by theoreticians are difficult to use by experimenters and cannot easily be tested with new data.

### Existing software

Many commercial and open source software packages have been developed to simplify such collaborative efforts and enhance code reuse. Chronux^1^, FIND (Meier et al., 2008), and the Spike Train Analysis Toolkit^2^ are algorithm toolboxes for MATLAB. However, while these toolboxes are open source, MATLAB itself is closed source and expensive. GNU Octave can be used as a free alternative, but it is not completely compatible with MATLAB and does not include all of its features. NeuroTools^3^ is an algorithm toolbox for Python. All of these libraries restrict users to a few supported file formats; data from other sources has to be converted before it can be used. FIND uses Neuroshare^4^ to support hardware vendor specific formats. Neuroshare is restricted to formats explicitly supported by the respective vendors and many formats only work on the Windows operating system. None of these libraries include a way of navigating and selecting data or using the analysis functions without programming.

Programs with a graphical user interfaces (GUI) provide such capabilities. There are various GUI programs available from hardware manufacturers and commercial vendors (e.g., Plexon^5^, Tucker-Davis Technologies^6^, or Nex Technologies^7^). However, the commercial programs are closed source, limited to the respective file formats of their creators, and only work on Windows. In addition, they cannot be extended by users. sigTOOL (Lidierth, 2009) is a MATLAB pogram with a graphical interface for data navigation that allows user extension. It supports several different data formats, but many of them only work in a 32-bit Windows environment and all data needs to be converted into a MATLAB format before it can be used by sigTOOL.

Non-commercial GUI programs that do not depend on MATLAB are rare. The program suite consisting of Dimstim, Spyke, and Neuropy (Spacek et al., 2008) supports data navigation and enables interactive work with a Python console, but does not support user extensions. The programs are very restricted in file format support and were written for the specific requirements of their authors. OpenElectrophy (Garcia and Fourcaud-Trocmé, 2009) includes a graphical interface and a library that users can use as a basis for their own analysis code. It has a strong focus on data management using a database: Many common file formats can be used but all data is loaded into a database before it can be accessed by OpenElectrophy. The GUI can only access tools provided by the developers (data visualization, oscillation detection, and spike sorting)—there is no interactive console and user extensions are limited to using the library for data access.

### Spyke viewer

**Figure 1:**
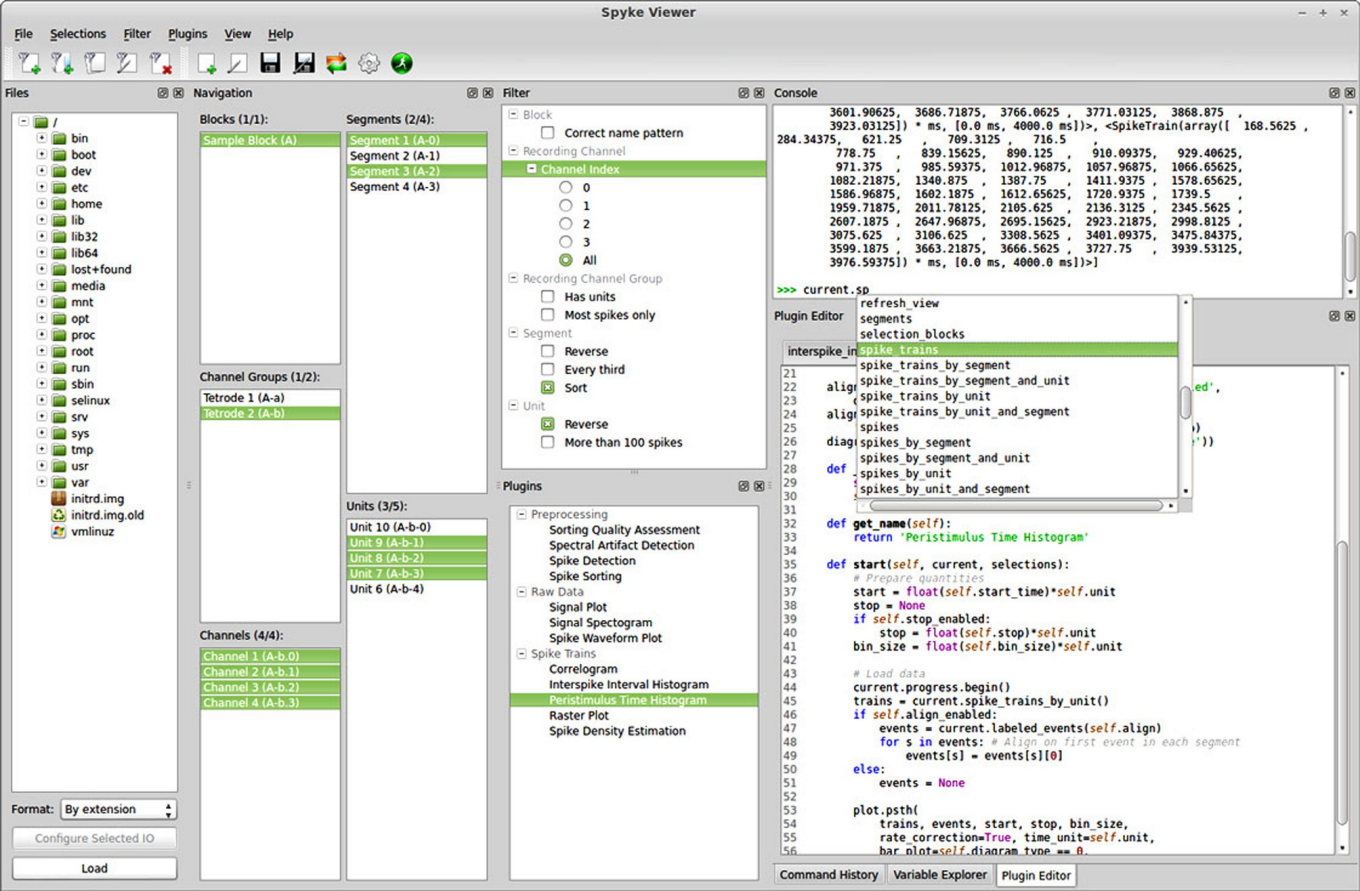
**Screenshot of the Spyke Viewer GUI.** The components of the program are arranged in dock elements. Most of the available docks are visible, the command history and variable explorer are tabbed together with the plugin editor. The dock layout is freely configurable and docks can be deactivated or detached to separate windows.

**Figure 2:**
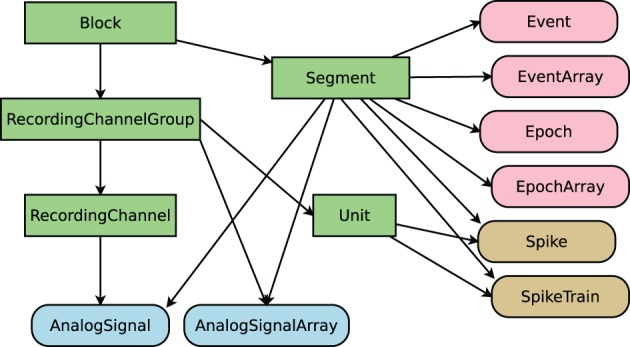
**Objects defined by Neo.** Rectangular items are container objects, data objects have rounded corners. Arrows indicate a “has zero or more” relationship. Colors of data objects denote to what containers they belong.

Spyke Viewer is an open source GUI application to browse, visualize and analyze data from electrophysiology experiments or neural simulations. It is completely written in Python and runs on Linux, OS X, and Windows with 32-bit and 64-bit architectures. Figure 1 shows a screenshot of the program. The GUI is organized in dock windows that can be configured into any desired layout. Spyke Viewer is built on top of the Neo library^8^, an object model for electrophysiology data implemented in Python. Neo is based on numpy^9^, the standard package for scientific computing in Python. In addition to numpy, Neo uses the quantities package^10^ to represent data together with its physical units in a concise and user-friendly manner. Figure 2 shows the objects that Neo defines and their relationships. Neo supports many file formats through custom Python classes, called Neo IOs. Currently, there are IOs for Neuroshare, Matlab, a custom HDF5-based format and more than 15 others, many of them vendor specific^11^.

With Spyke Viewer, users can load any data format supported by Neo and browse and select the contents. They can use an integrated Python console to interactively work with subsets of the loaded data, using the whole Python scientific stack and custom libraries. Python is also well suited to interface to other languages such as C/C++, Matlab, or R using appropriate modules.

Spyke Viewer is designed to be flexible and extensible. When working with large datasets, users often only care about a particular subset. A filtering system allows users to hide Neo objects they are not currently interested in. Filters are user defined Python functions and are manipulated directly from the GUI.

In addition to the console, analysis plugins provide the visualization and analysis capabilities of Spyke Viewer. Analysis plugins are Python classes and can be created and edited in the GUI or using an external editor. They perform operations on the selected data using arbitrary Python code (so they can use Python libraries or even other languages) and can range in complexity from showing the number of currently selected spikes to complex multi-stage analyses, possibly with their own GUI.

Many users want to analyze data stored in a non-standard format. Using IO plugins, they can use Spyke Viewer with their existing data and do not need to convert it. If they want to share data with users of other tools, Spyke Viewer provides data export to multiple standard formats using Neo.

Because all data in Spyke Viewer is internally represented as Neo objects, analysis plugins work with data from any source without code changes. This standardization enables convenient sharing of algorithms and easier replication of results. It also facilitates testing algorithms on different data sets. On the Spyke Repository website, users can find existing plugins and share their own contributions.

Spyke Viewer and Neo are focused on local field potential and spike data. Although data from other sources such as electroencephalography (EEG) can be processed, they are not explicitly supported: Neo does not include IO classes for many EEG data formats and the plugins included with Spyke Viewer do not offer specialized EEG plots.

## Software architecture

Spyke Viewer consists of two Python packages: *spykeutils* and *spykeviewer*. *spykeutils* contains components related to data management, the core of the plugin system, analysis algorithms and plotting functions. It is independent of *spykeviewer*, has fewer dependencies and no graphical interface, so it can be installed on servers without a graphical environment (though interactive plots will not work in this case). Using *spykeutils*, plugins can be run on a compute server. The *spykeviewer* package contains the GUI program, including the navigation and filtering systems and plugin management. Here, we will mostly ignore the distinction and refer to both packages as Spyke Viewer.

The code of the most recent development version and all previous stable releases is available on GitHub^12^. The GitHub web page also include an issue tracker for bug reports and feature requests. Using the Git version control system together with GitHub facilitates collaborative development and gives users of the program a convenient way to contribute code changes.

Software quality and documentation are an important aspect of software development that receive little attention in many scientific software packages. Spyke Viewer currently has almost 200 unit tests to ensure that new features do not break existing functionality. We use continuous integration (Zaytsev and Morrison, 2012) to get immediate feedback on code changes. In the same way, our documentation is automatically updated and available online for all releases and the current development version^13^.

### Data selection

The first step in working with any dataset of sufficient size is selecting the particular subset of the data one wants to investigate. In Spyke Viewer, a selection describes a connected subgraph of Neo container objects (see Figure 2). On the top level, it consists of a list of block objects with information on where they can be found (e.g., a path in the file system). Objects further down in the hierarchy are then referenced by their relations to higher objects (e.g., the third segment of a block). In this way, a selection is self-contained: it can be saved to disk and includes all necessary information to load the container and data objects it references. Because selections do not contain the data itself, they use little memory. In the Spyke Viewer GUI, users can create selections spanning data from multiple sources in an intuitive way using the navigation dock. Multiple selections can be managed from the GUI and saved to or loaded from human readable JSON files.

Usually, only a semantically defined subset of the data is relevant at a given time, for example all Segments that were recorded while a particular stimulus was presented. As it can be cumbersome to manually select such subsets from large datasets, Spyke Viewer includes a filtering system that determines which objects are shown in the navigation dock. A filter is a Python function that operates on a list of container objects. It returns which items are to be displayed and in what order. Multiple filters can be linked into filter chains that are processed in order. For convenience, it is also possible to define filters that only act on single objects and return whether the object should be displayed. We have decided to use Python functions to give maximum flexibility to the user because datasets are heterogeneous and will often need custom filters. Because Python has a syntax close to natural language and most filters are simple, it is easy to create them. For example, a filter operating on single objects to only show blocks that contain at least 10 segments:

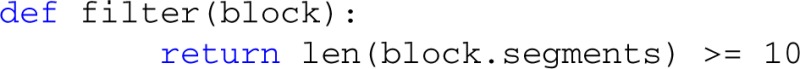



When this filter is active, Spyke Viewer calls this function for each loaded block. The function then returns whether the list of segments in the block contains at least 10 items. Blocks for which the function returns false are not shown in the navigation dock.

### Data provider

Once users have selected data of interest, they need a way to access this data. In Spyke Viewer, this is realized by the DataProvider class. Each DataProvider object encapsulates a selection and provides a large number of methods to query the selected Neo objects. This additional layer of abstraction between users and data has several advantages:

- Convenience for common usage patterns: For example, getting all selected analog signals ordered by recording channel requires just one method call.
- Control over when the data is acquired: Spyke Viewer supports transparent lazy loading by initially only reading container objects when a file is opened and then automatically loading the missing data as needed during calls to DataProvider methods.
- Specialization: DataProvider itself is an abstract base class. Spyke Viewer currently contains two implementations: one uses selections in memory as described above (e.g., loaded from a selection file), the other always uses what is currently selected in the GUI. Other implementations are possible: For example, database access can be implemented more efficiently with a DataProvider subclass than is currently possible using a Neo IO.

### Console

One way to interact with the DataProvider objects is through an interactive console. Spyke Viewer provides two options: An internal console and IPython. The internal console is implemented using components from *spyderlib*^14^ and integrates completely into the GUI as a dock. Spyke Viewer also includes a command history and a graphical variable explorer for the internal console in separate docks.

IPython is a popular shell in the scientific Python community (Perez and Granger, 2007). We have embedded an IPython kernel into Spyke Viewer, allowing external IPython consoles to attach to the program. The IPython consoles can then be used in the same way as the internal console.

Two important objects are defined in the console environment: current, a DataProvider object representing the presently selected objects in the GUI, and selections, a list of DataProvider objects representing stored selections. These objects, together with common scientific Python packages such as *scipy* and *matplotlib*, enable efficient exploratory data analysis.

### Plugins

Extensibility is a central design goal of Spyke Viewer. It is achieved with a flexible plugin architecture that allows easy creation of new plugins that can be started directly from the GUI. The core of the plugin system is implemented in the *spykeutils* package, so plugins can also be executed without the Spyke Viewer program.

An important decision for every plugin architecture is how plugins in the file system are discovered and integrated. In order to keep the plugins as simple as possible, Spyke Viewer scans a list of user defined directories for Python files. The directories are scanned recursively and subdirectories are represented in the GUI, helping to organize plugins. In each of the Python files, Spyke Viewer searches for plugin classes. A plugin class inherits from an abstract plugin base class and implements two methods: get_name returns the name of the plugin used in the GUI, start contains the code to run when the plugin is executed.

**Figure 3:**
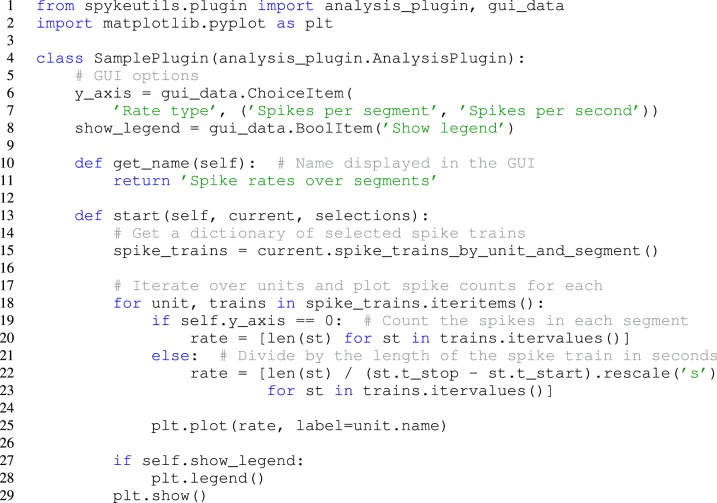
**A complete plugin that plots spike rates over multiple segments.** It includes two options that can be adjusted in the Spyke Viewer GUI.

Spyke Viewer includes a Python editor that can be used to create and edit plugins directly from the GUI. Like the internal console, the editor uses components from *spyderlib* and supports syntax highlighting, autocomplete and Python docstrings as tooltips. However, it is also possible to edit plugins in an external editor. The plugins are refreshed at runtime, so it is not necessary to restart Spyke Viewer when plugins change. Figure 3 shows an example of a complete plugin for Spyke Viewer that uses *matplotlib* to plot spike rates over time, with one data point per segment. This plugin and its creation are described in more detail in the next section.

Many analysis algorithms or plots have various parameters. Users should not have to change the plugin code every time they want to change a parameter, so Spyke Viewer uses the *guidata* package^15^, to make creating graphical options very simple. See Figure 3, lines 6–8, for an example of two such options. Because *guidata* requires graphical packages, it is wrapped in *spykeutils* so plugins behave as expected when *guidata* is not available.

Analyses can require long execution times and may produce large results which are reused, e.g., in later stages of a processing pipeline. For convenience, Spyke Viewer provides an interface to save results connected to a parameter set (by default the current selection and plugin parameters) to HDF5 files. The resulting files are indexed using this information so they can be retrieved quickly. Because plugins are regular Python code, they can also employ arbitrary other persistence schemes such as saving results to a database. In addition, the Spkye Viewer API includes functions to execute plugins. Together, these features enable the creation of simple workflows through plugins calling and retrieving data from other plugins or the creation of dispatcher plugins that can implement more complex workflow scenarios.

Spyke Viewer provides multiple ways in which plugins can be executed. The default method starts the plugin in the same process as the program itself. This has the advantage of shared memory: the plugin can access all data that has been loaded into memory during execution of Spyke Viewer. This is the fastest method, but it has a drawback: the GUI can not be used while the plugin is working, except for progress display.

Another method is to start the plugin in its own process. In this case, the selection, plugin code and parameters are serialized and sent to a new process. The new process then creates the necessary DataProvider objects and starts the plugin. Because the plugin execution is independent of Spyke Viewer, the required data needs to be loaded in the new process. However, the GUI is unaffected by the plugin execution and multiple plugins can be run at the same time.

Finally, the execution of plugins can be completely decoupled from Spyke Viewer. A small Python script loads a plugin, stored selections and additional context information and then executes the plugin. Spyke Viewer can be configured to save the necessary information instead of directly starting a plugin in a new process. This can be used for reproducibility, batch processing or execution on a server.

Despite the large number of file formats that Spyke Viewer supports through Neo, there are many custom data formats that Spyke Viewer cannot load directly. In order to use Spyke Viewer with such data, users have two choices: they can either convert the data to a standard format that Neo can read, or they can write an IO plugin. These plugins are implemented as Neo IO classes that load the format into the Neo object hierarchy. They inherit from neo.io.BaseIO, the abstract base class also used for all regular Neo IO classes. IO plugins are discovered in the same way as regular plugins when placed in one of the plugin directories. In addition to reading, IO plugins can also support writing data. Spyke Viewer offers data export using all available Neo IO classes and IO plugins that support writing.

**Figure 4:**
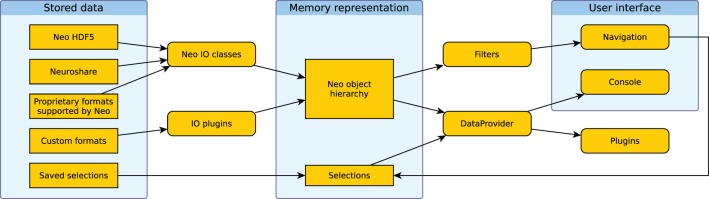
**Illustration of the data flow in Spyke Viewer.** Rectangles with sharp corners represent data on disk or in memory, rounded corners indicate Spyke Viewer components.

Figure 4 shows how the different components of the system work together. Data is loaded into the Neo object hierarchy using Neo or IO plugins. The active filters determine which objects are displayed in the Navigation dock in the GUI. Users then select a subset of these objects. The selection from the navigation dock is used to create a DataProvider object. In addition, multiple selections can be stored and are used to create further DataProvider objects. These objects can then be used to access the selected data in the integrated console, in IPython or in analysis plugins.

## Usage

### Installation

Python is well suited to developing both scientific algorithms and large scale graphical applications such as Spyke Viewer thanks to the robust language design and availability of mature libraries for scientific computing, plotting, and GUI development. As an interpreted language, Python also lends itself cross-platform development. By using PyQt as an interface to the Qt GUI library, the graphical interface runs and looks native on Linux, OS X, and Windows.

However, while developing a Python program that runs on different platforms works well, distributing the program can be difficult. The standard way to distribute a Python application is the Python Package Index (PyPi)^16^. However, PyPi requires an existing, properly configured Python installation. Because Spyke Viewer also targets users with little or no Python experience, we have tried to find further options apart from PyPi.

For Debian-based Linux distributions (e.g. Ubuntu), the NeuroDebian project (Halchenko and Hanke, 2012) provides a large number of neuroscience research software packages. We maintain Spyke Viewer packages in NeuroDebian, so Debian users can install the program with their package manager using the NeuroDebian repository.

For Windows and OS X users, we use PyInstaller^17^ to create binary packages. PyInstaller collects the Python interpreter, the Python files from Spyke Viewer, and all required libraries into one package that can be used without a regular Python installation. While these packages are easy to use, they have some drawbacks: they are larger than the source package provided by PyPi and users cannot simply install additional libraries as in a regular Python installation.

### Filters

**Figure 5:**
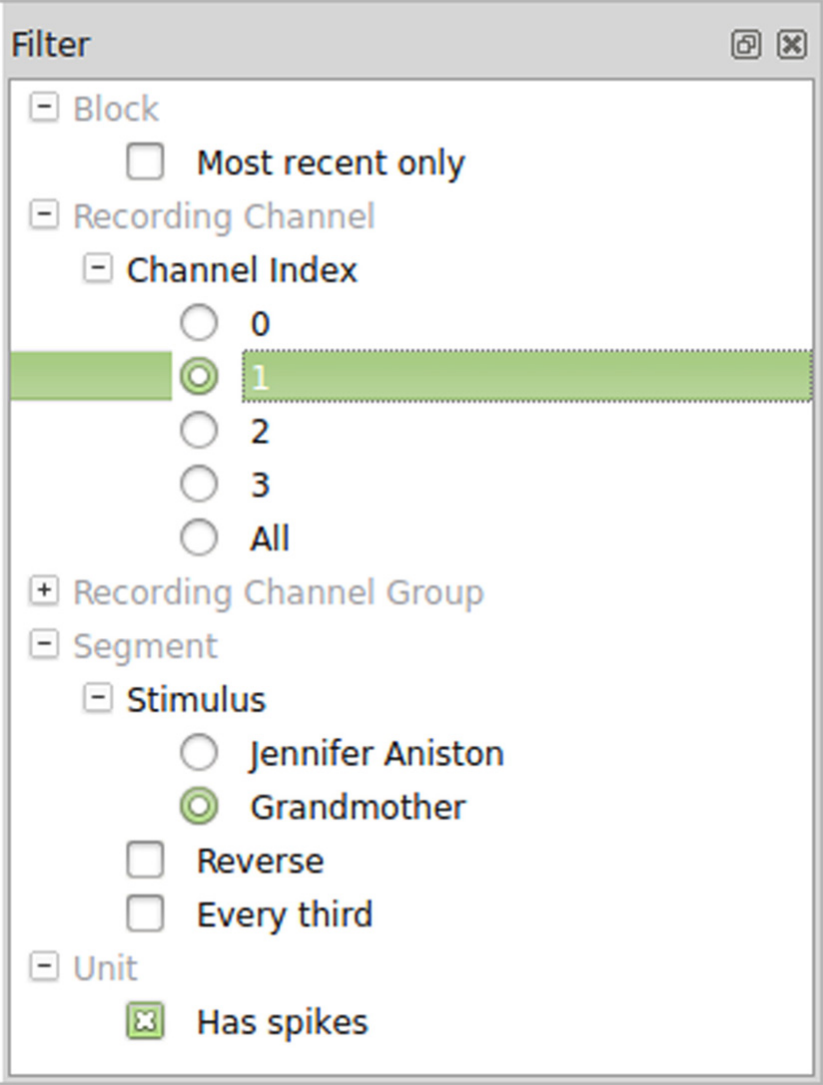
**The filter dock allows users to order and activate filters**.

Once Spyke Viewer is installed, users can load supported files and explore their contents. For larger datasets, it is often useful to hide non-relevant parts of the data using the plugin system. The decision to implement filters as Python functions maximizes flexibility but could be less comfortable to use than a more restricted solution. To alleviate this issue, Spyke Viewer integrates all aspects of filter management into the GUI. In the filter dock (see Figure 5), users can toggle filter activation, organize them into groups and order them using drag and drop.

**Figure 6:**
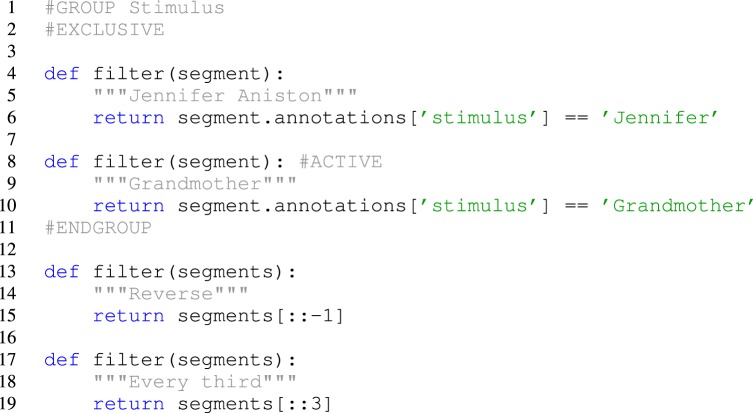
**Code for segment filters in Figure 5.** When filters are edited directly in the GUI, only the function body has to be written.

Filters can be created or edited using a GUI editor. They are saved as Python files (with annotations in comments) so they can also be edited externally and shared with other users. Figure 6 shows such an annotated Python file for the segment filters in Figure 5. The first two filters check the annotations of a segment for a particular stimulus and are part of an exclusive filter group named “Stimulus.” Exclusive groups can only include a single active filter. The other two filters are not mutually exclusive and operate on the complete list of segments instead of single items. They reverse the list or only admit every third item, respectively.

### Included plugins

**Figure 7:**
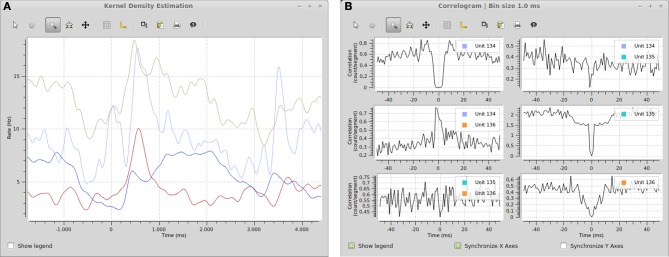
**(A)** Spike density estimation of four units. Time 0 in the plot corresponds to an experimental stimulus event. The kernel bandwidths for the different neurons were optimized independently. **(B)** Autocorrelograms and cross-correlograms for three units. When plots include multiple subplots, the suplot axes can be synchronized independently.

The Spyke Viewer package includes several plugins for common plots. See Table 1 for the full list. Figure 7 shows examples of plots produced by two of these plugins. They use the *guiqwt* plotting library^18^. Compared to *matplotlib*, which can be considered the standard library for plotting in Python, *guiqwt* offers better performance and more tools for interactive use. For publication-quality plots, *matplotlib* is preferable and available in Spyke Viewer from the console and in plugins.

**Table 1:** **Standard plotting plugins included with Spyke Viewer**.

**Plugin**	**Description**
Correlogram	Creates subplots with autocorrelograms and cross-correlograms for selected units.
Interspike interval histogram	Displays the interspike interval distribution for selected units.
Peristimulus time histogram	Shows the peristimulus time histogram (PSTH) for selected units. Spike trains can be aligned on an event before calculation.
Raster plot	Displays the spike times for multiple units in one segment or multiple segments and one unit.
Signal plot	Shows analog signals for selected recording channels in subplots or one shared plot. Optionally overlays events, epochs, spike times and spike waveforms.
Signal spectrogram	Creates subplots with spectrograms (time-frequency histograms) for selected recording channels.
Spike density estimation	Shows a kernel density estimation of spike rates for selected units. Spike trains can be aligned on an event before calculation. Optionally finds the optimal kernel bandwidth (Shimazaki and Shinomoto, 2010).
Spike waveform plot	Displays spike waveforms for selected units. Offers various configurations to split recording channels or units into subplots.

The included plugins enable quick exploratory analyses of both analog signals and spike data for datasets of any size. For example, the peristimulus time histogram (PSTH) and spike density estimation plugins can show average firing rates during experimental trials. Together with the filter system (e.g., the stimulus filters described above), these plugins could be used to compare the influence of various stimuli on the firing rate of neurons. Figure 7 shows an example of a spike density estimation plot comparing the influence of a stimulus on four neurons.

### From console to plugin

After the initial exploration of the data, users usually want to perform custom analyses. We now illustrate with a simple example how Spyke Viewer can be used to create a plot that is not available with the included plugins. Consider the case where users have analyzed the stimulus dependent firing rate changes and are now interested in how the activity of a neuron evolves over a longer time, spanning many segments of a recording. They want to visualize the spike count of a selected unit over segments. After selecting the unit and segments of interest, they use the internal console to query the currently selected spike trains:


>>> trains = current.spike_trains()


The trains variable could now be inspected using the variable explorer dock: it contains a list of Neo spike train objects. Now, the users can count and plot the number of spikes in each spike train. Because *matplotlib* is imported by default in the console under the name plt, this can be done in one line:

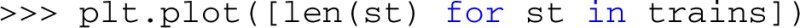



This code uses a Python list comprehension to concisely define the list of values to be plotted: The length of a spike train (i.e., the number of spikes) for each spike train in the variable trains. After examining a few neurons in this way, the users might decide that this is a useful plot that they want to keep with convenient access. They might also want to add some additional features, such as multiple neurons in the same plot for easy comparison. These requirements are best satisfied by writing a plugin.

When writing a new plugin in the GUI, Spyke Viewer creates a template with code for a minimal functional plugin. The users can import *matplotlib* and copy the two lines they used to create the plot from the console history into the start method of the template plugin. Figure 3 shows the final plugin after adding the ability to plot multiple units. This plugin also has two graphical options that control if the number of spikes is shown as a count per segment or a rate in Hertz and if a legend is included in the plot. When the plugin is saved, it immediately appears in the Spyke Viewer plugin list and is ready for use.

### Startup script

The startup script offers a way for users to customize Spyke Viewer. The startup script is executed whenever Spyke Viewer is started, before plugins or data are loaded. It can be used to set configuration options defined in the Spyke Viewer API, change global parameters like the colors used in plots or even manipulate the GUI itself. The following code would disable automatic loading of previously opened files when starting the application and increase the font size in the console:

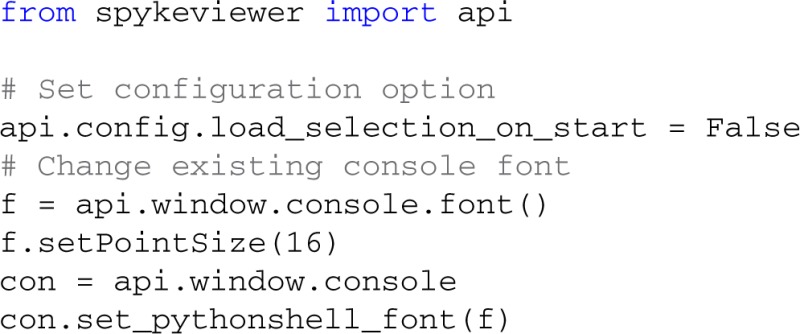



## Extension repository

**Figure 8:**
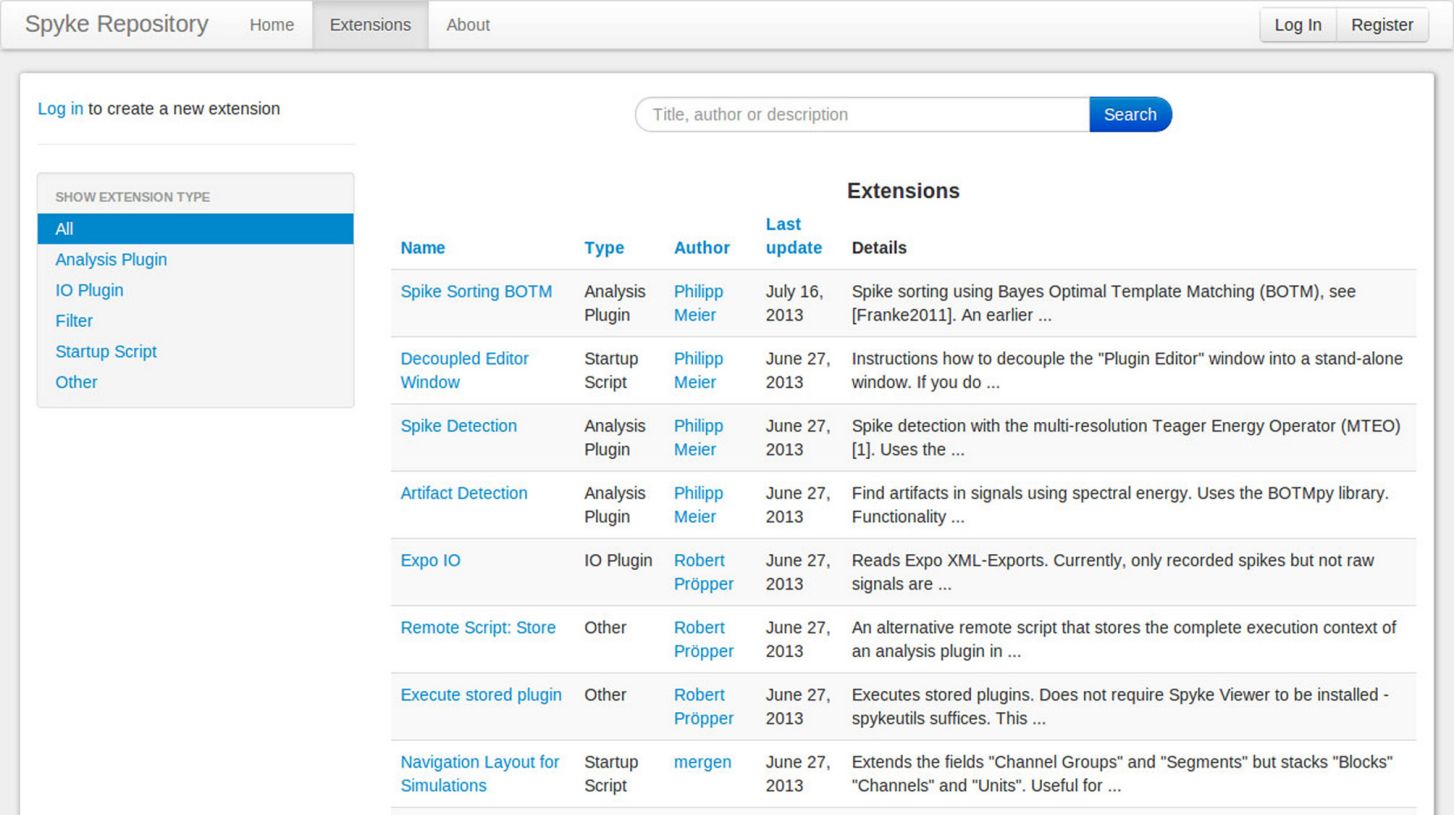
**Extension list on the Spyke Repository website**.

The Matlab File Exchange^19^ is a popular resource for MATLAB users to find and share code. Currently, there is no equivalent for Python users. Although PyPi works well for sharing libraries, it requires users to create setup scripts and the libraries are installed in a fixed directory, so it is not suited for plugins or other Spyke Viewer extensions. To foster sharing and grow a community around Spyke Viewer, we have created the Spyke Repository: a website where users can upload their Spyke Viewer extensions and search, download or comment existing extensions from others. Figure 8 shows a screenshot of the extension list page. The site is hosted at the G-Node (Herz et al., 2008) and can be reached at http://spyke-viewer.g-node.org.

Some of the early content on the site are scripts used to execute plugins on servers. Other examples include plugins for artifact detection, spike detection, automatic spike sorting (Franke et al., 2010), and an IO plugin for Expo^20^ files.

## Discussion

We have described Spyke Viewer, a flexible and extensible platform for analyzing data from electrophysiology experiments and computer simulations. It is based around the common object model provided by the Neo library. A central design goal was to restrict the user as little as possible while providing convenient functionality for selecting, accessing, visualizing, and analyzing data. The most important feature in this respect is the plugin system that facilitates exploratory data analysis and algorithm development, and includes features for reproducibility and running time consuming analyses. No previous work has offered the combination of open source, convenience, and flexibility that Spyke Viewer gives its users.

Thanks to its GUI, Spyke Viewer provides a wide range of functionality for users who do not develop algorithms themselves. It includes several plugins for common visualizations used in neuroscience. Plugins developed by others can be applied to different datasets without writing code, considerably reducing the effort of sharing data and code between scientists and laboratories. The Spyke Repository website lists extensions created by users and allows developers to share their extensions with the community. Python has a large community of scientific users, including many neuroscientists. These researchers have produced a wide variety of useful Python packages for data analysis and visualization. Thanks to the flexibility Spyke Viewer offers to its users, these packages can be used from the internal console or turned into analysis plugins. By leveraging existing libraries and tools, small plugins can perform complex analysis steps such as spike sorting. Since Python also works well as a glue language, Spyke Viewer has the potential to unify existing tools by providing a central GUI from which independently developed analyses can be launched, regardless of whether they exist as Python libraries, in other programming languages or as standalone programs.

### Conflict of interest statement

The authors declare that the research was conducted in the absence of any commercial or financial relationships that could be construed as a potential conflict of interest.

## Footnotes

^1^http://www.chronux.org/

^2^http://neuroanalysis.org/

^3^http://neuralensemble.org/NeuroTools/

^4^http://neuroshare.sourceforge.net/

^5^http://www.plexon.com

^6^http://www.tdt.com

^7^http://www.neuroexplorer.com

^8^http://neuralensemble.org/neo/

^9^http://www.numpy.org

^10^http://packages.python.org/quantities

^11^Full list at http://neo.readthedocs.org/en/latest/io.html

^12^https://github.com/rproepp/spykeviewer

^13^http://spyke-viewer.readthedocs.org/

^14^https://code.google.com/p/spyderlib

^15^https://code.google.com/p/guidata

^16^http://pypi.python.org/

^17^http://www.pyinstaller.org

^18^http://code.google.com/p/guiqwt

^19^http://www.mathworks.com/matlabcentral/fileexchange/

^20^https://sites.google.com/a/nyu.edu/expo
